# Implementation of the HealthKick intervention in primary schools in low-income settings in the Western Cape Province, South Africa: a process evaluation

**DOI:** 10.1186/s12889-015-2157-8

**Published:** 2015-08-22

**Authors:** Anniza de Villiers, Nelia P. Steyn, Catherine E. Draper, Jillian Hill, Lucinda Dalais, Jean Fourie, Carl Lombard, Gerhard Barkhuizen, Estelle V. Lambert

**Affiliations:** Non-communicable Diseases Research Unit (NCDRU), South African Medical Research Council (SAMRC), Francie van Zijl Drive, Tygerberg, 7505 South Africa; Division of Human Nutrition, University of Cape Town Medical Campus, Anzio Road, Observatory, 7925 South Africa; Division of Exercise Science and Sports Medicine, Department of Human Biology, Faculty of Health Sciences, University of Cape Town, PO Box 115, Newlands, 7725 South Africa; Biostatistics Unit, SAMRC, Francie van Zijl Drive, Tygerberg Cape Town, 7505 South Africa; Western Cape Education Department (WCED), Cape Town, South Africa

## Abstract

**Background:**

The HealthKick intervention, introduced at eight primary schools in low-income settings in the Western Cape Province, South Africa, aimed to promote healthy lifestyles among learners, their families and school staff. Eight schools from similar settings without any active intervention served as controls.

**Methods:**

The Action Planning Process (APP) guided school staff through a process that enabled them to assess areas for action; identify specific priorities; and set their own goals regarding nutrition and physical activity at their schools. Educators were introduced to the APP and trained to undertake this at their schools by holding workshops. Four action areas were covered, which included the school nutrition environment; physical activity and sport environment; staff health; and chronic disease and diabetes awareness. Intervention schools also received a toolkit comprising an educator’s manual containing planning guides, printed resource materials and a container with physical activity equipment. To facilitate the APP, a champion was identified at each school to drive the APP and liaise with the project team. Over the three-years a record was kept of activities planned and those accomplished. At the end of the intervention, focus group discussions were held with school staff at each school to capture perceptions about the APP and intervention activities.

**Results:**

Overall uptake of events offered by the research team was 65.6 % in 2009, 75 % in 2010 and 62.5 % in 2011. Over the three-year intervention, the school food and nutrition environment action area scored the highest, with 55.5 % of planned actions being undertaken. In the chronic disease and diabetes awareness area 54.2 % actions were completed, while in the school physical activity and sport environment and staff health activity areas 25.9 and 20 % were completed respectively. According to educators, the low level of implementation of APP activities was because of a lack of parental involvement, time and available resources, poor physical environment at schools and socio-economic considerations.

**Conclusions:**

The implementation of the HealthKick intervention was not as successful as anticipated. Actions required for future interventions include increased parental involvement, greater support from the Department of Basic Education and assurance of sufficient motivation and ‘buy-in’ from schools.

## Background

Several studies undertaken with schoolchildren in South Africa have implied that unhealthy diets [1–3] and physical inactivity [4–6] present an emerging public health challenge. Furthermore, strong evidence exists that these unhealthy practices track into adulthood [7]. Since South Africa is facing a growing burden of non-communicable diseases (NCDs) among its adult population, it is incumbent on health policy makers and educators to consider health promotion interventions among children and adolescents. According to the World Health Organization, NCDs are estimated to have attributed to 28 % of the burden of disease in South Africans [8]; however, research has indicated that 80 % of this burden can be prevented by limiting exposure to modifiable risk factors, such as unhealthy diets and physical inactivity, tobacco use, and alcohol abuse [9]. The National School Health Policy and Implementation Guidelines (since replaced by the Integrated School Health Policy [ISHP]) [10] existed in South Africa at the start of the HealthKick project but with inadequate attention to NCD risk factor prevention in the guidelines, the HealthKick project provided an opportunity for investigating the feasibility of addressing these risk factors through the school environment.

In 2007, as part of the formative phase of the study presented in this paper, a situation analysis survey was undertaken in a representative sample of 100 primary schools situated in low-income communities in one urban and one rural education district, in the Western Cape Province, South Africa [11]. This study provided valuable information regarding healthy lifestyles of learners, parents and educators from the principals’ point of view. These principals identified the top three health priorities in learners, parents and educators. In learners these were an unhealthy diet (76 %), lack of physical activity (50 %), and being underweight (47 %). Top health priorities identified for parents were substance abuse (91 %), tobacco use (57 %), and an unhealthy diet (47 %). Those identified for educators were lack of physical activity (33 %), NCDs (24 %), and being overweight and having an unhealthy diet (12 %) [11]. Of the educators surveyed, 31 % were found to be overweight, 47 % were obese, 56 % were hypertensive, 80 % used tobacco, 77 % were inactive, and 30 % had high blood cholesterol levels [12].

Direct observation of the physical activity and nutrition environment at these schools was also done. Principals’ perceptions, the health risk assessment of educators, and the observed practices at surveyed schools, clearly pointed to the need for healthy lifestyle promotion for learners, parents and educators. With this in mind, the research team set out to plan and develop the HealthKick (HK) intervention as described in an earlier paper [13].

The overall aim of the HK intervention was to promote a healthy lifestyle for the general well-being of primary school learners, their families and educators; and to prevent NCDs. Specific objectives were to promote healthy eating habits; to increase regular participation in physical activity and to develop an environment within the school and community that promotes and facilitates these objectives through an action planning process (APP). The aim of the present study is to describe the implementation of the HK programme, and identify some of the barriers that may have influenced the process. The implementation and evaluation of the curriculum component of the intervention is described in greater detail elsewhere [14].

## Methods/Design

### Study population

The HK study comprised sixteen eligible schools selected from the representative sample of 100 primary schools surveyed in two conveniently selected educations districts (one urban and one rural) in the Western Cape Province of South Africa during the formative phase of the study. The number of schools included in the intervention study was predetermined by the study protocol and the available budget. Eligibility for participation in the study was determined by the formative findings and included (i) whether the principal expressed the need for a health promotion programme to be implemented in the school (ii) the availability of at least one grass field or access to community sport facilities (iii) the presence of a shop or vendor selling food items at the school (iv) unhealthy diet and lack of physical activity among learners and teachers selected as a top health priority by the school principal (v) the view of the education district level managers of the potential of schools to effect changes, subjectively taking into account functionality (*i.e.* functional School Based Support Team; School Management Team), ethos (co-operation, will, inclination) and viability of school (*e.g.* results/performance of schools), (vi) distance from the research office (not more than 105 min’ drive) (vii) school size (schools with less than 50 grade 4 learners were excluded). The list of 35 eligible schools were stratified by (i) site (urban versus rural) (ii) poverty level (quintile 1 and 2 versus quintile 3 schools), and (iii) school size (schools with less than 100 grade 4 learners versus schools with more than 100 grade 4 learners). This resulted in seven distinct strata. Due to the small number of schools in each stratum it was decided to make use of manual allocation and four schools were randomly selected from the largest strata (nine schools) and two schools each from the smaller strata by drawing lots. The project coordinator (AdeV) together with a field coordinator (LD) was responsible for the random allocation. The process involved drawing the names of schools typed on folded white paper of exactly the same shape and size from a container. Consent from the principals of the selected schools was obtained. There was one refusal in the largest stratum and this school was replaced randomly by the same method. The consolidated schools were then randomised to intervention and control arms within each stratum using the same methodology and with the person doing the selection blinded to whether the selected school would be allocated to intervention or control. The allocation sequence was decided on by the project coordinator before the selection took place.

### Intervention components

**Fig. 1:**
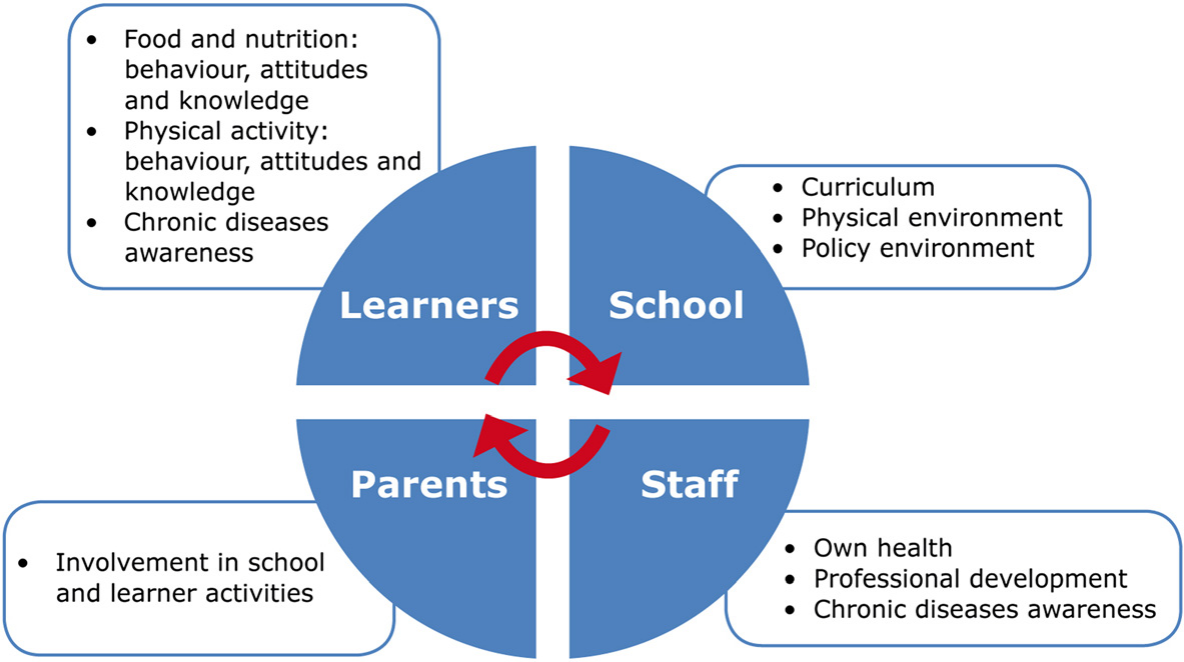
Nutrition- and physical activity-related goals centred in various areas, viz., learners, parents, teachers and staff

**Fig. 2:**
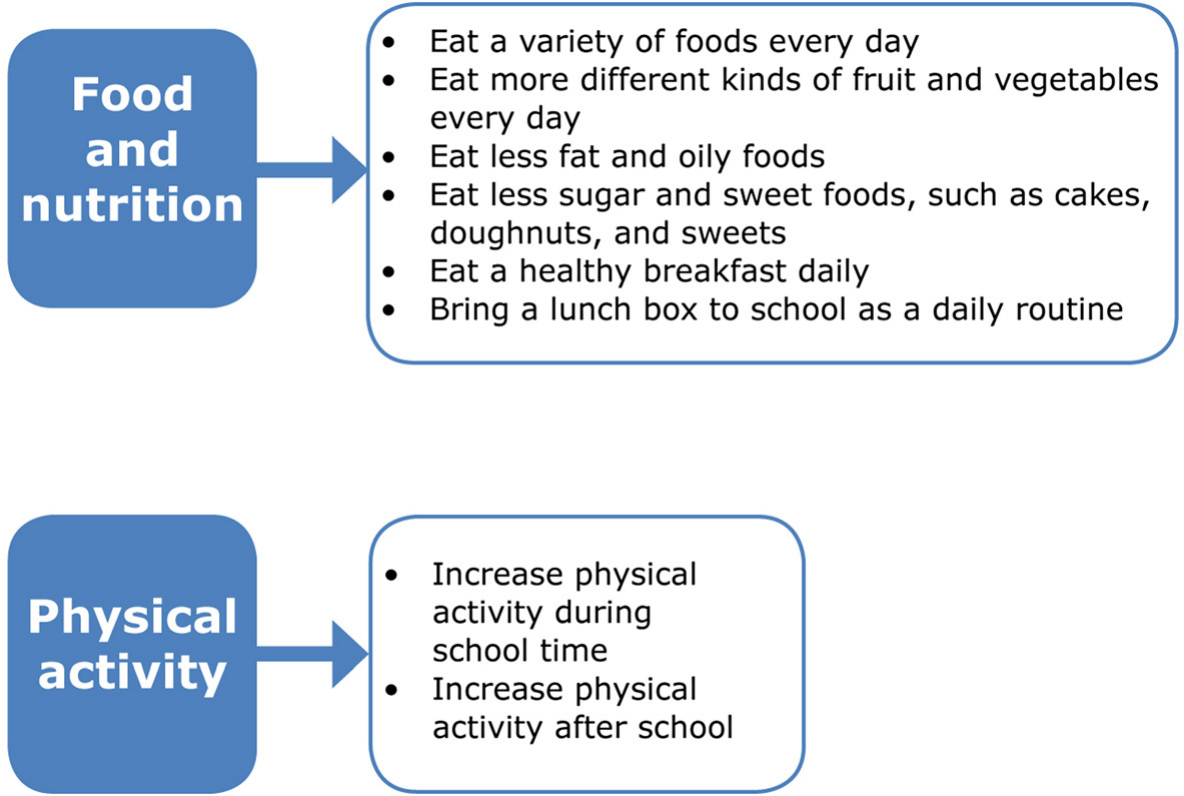
Behaviour outcomes for learners in the HK programme as identified during IM

HK intervention behavioural outcomes leading to a healthy diet and physical activity were developed using the intervention mapping (IM) approach [15]. This process was closely aligned with the South African food-based dietary guidelines [16, 17] and physical activity guidelines for schools to ensure compliance with national dietary and physical activity objectives for South Africans. During the IM process, different aspects that were identified as priorities to address in the intervention are summarised in Fig. 1, while behaviour outcomes for the children are shown in Fig. 2. The APP based on Action Schools British Colombia (AS! BC) Planning Guide for Schools [18, 19] and the Centres for Disease Control’s School Health Index [20] formed the basis of the HK programme, of which the development is described in greater detail elsewhere [13].

The preparation for the formal implementation of HK in the intervention schools started in 2008 with sessions guiding the staff from a specific school through a process that enabled them to assess areas for action, identify specific priorities, and set their own goals regarding nutrition and physical activity at their school. During these sessions four action areas were covered. These included the school food and nutrition environment; school physical activity and sport environment; staff health; and chronic diseases and diabetes awareness (see Fig. 1) and were later consolidated in the booklets included in the educator’s manual described below. To facilitate and drive the formal implementation of the APP, a champion (teacher) was identified at each school and they were encouraged to liaise with the project team whenever they required assistance. The formal intervention started in 2009 and schools were given the following intervention package/toolkit:

i) An “educator’s manual” which contained an APP guide, a booklet for each action area containing guidelines for prioritising action as well as strategies to address identified priorities; the South African food-based dietary guidelines; a poster listing the behaviour outcomes desired for the children; a poster for listing planned actions; and in 2011 a healthy lifestyle guide for teachers was included.
ii) A container of physical activity resources, *e.g.* skipping ropes, chalk for playground markings, soccer balls.
iii) A resource box with printed materials relating to a healthy lifestyle and its role in the school curriculum.
iv) A curriculum support manual integrating the HK goals with the existing Life Orientation curriculum, developed by an expert in a format familiar to educators.
v) In 2011, a series of mobile phone messages to parents, based on the HealthKick behaviour outcomes was added to the intervention package. (The implementation and outcome of this mobile phone intervention will be reported in a separate paper).

Optional intervention support was offered to the intervention schools in all four action areas during the three years of the intervention. The support took the form of structured activities by the research team to broaden the staff’s knowledge and skills around actions to support a healthy lifestyle. Furthermore, the research team kept in regular contact with the schools who were encouraged to call for assistance/support from the research team at any time.

Principals at schools in the control arm received a booklet with “tips” for healthy schools and a guide to resources that could be accessed to assist in creating a healthier school environment. No further engagement took place between the research team and these schools except for the annual learner and environmental survey.

### Evaluation instruments and data analysis

Over the three-year period a record was kept of all activities/events planned by intervention schools and the number that were actually carried out. Activities were logged in an Excel spread sheet in the action areas under which they took place and percentages were calculated based on either the total number of schools or the total activities planned.

Data from the situational analysis were used as baseline information and an adapted version of the principal questionnaire and observational schedule used during the formative assessment which was completed annually at all 16 schools. On completion of the intervention, focus group discussions were held with school staff at each school, while principals and school staff involved with the school nutrition programme, tuck shops and vegetable gardens were interviewed using semi-structured questionnaires. The interviews and focus group discussion were conducted by members of the research team. Team members served as both implementers and researchers as available resources did not allow for two teams. Although this could be considered a limiting factor, only two team members conducted all interviews and focus groups and an administrative person kept record of all intervention activities. A good rapport between school staff and the research team allowed for a free flow of thoughts during the qualitative evaluation.

All interviews and focus group discussions were recorded and transcribed, and the data managed with ATLAS.ti Qualitative Data Analysis Software (Scientific Software Development GmbH, Berlin, Germany). Initial data analysis involved coding the focus group data as group interviews (*i.e.* similar responses coded only once per group). From this analysis, three code categories were identified as important for evaluation and were subsequently used to code all interviews as described above. Different themes were then identified in each category and the number of codes in each theme was used to rank the themes from salient to least mentioned. Two team members (AdeV and JH) were responsible for analysing the data and agreement on codes and themes were reached at the end of each stage of the analysis process [21].

### Ethics

Ethical approval for this study was obtained from the Human Research Ethics Committee in the Faculty of Health Sciences, University of Cape Town (HREC REF: 486/2005). In addition, approval for intervention in primary schools was obtained from the Western Cape Education Department. Written consent was obtained from all participants in the interviews and focus groups. Written parental consent was obtained for learners participating in the research.

## Results

Table 1 shows some of the pre and post intervention characteristics of the intervention and control schools. Concerning the school nutrition environment, 11 of the 16 schools had shops selling food and beverages, and five of them had vendors selling food items within close proximity. Two of the intervention schools started with a shop at the school during the intervention. One of these schools requested intervention support in the form of training for the shop keeper and managed to incorporate fruit in the list of items that being sold. These findings are incorporated in the number of actions presented in Table 2. Table 1 also reports on a rise in the number of vegetable gardens in especially the intervention group. At the start of the intervention only one intervention school had a vegetable garden and at the end four gardens were established. With regard to staff health, it is clear from Table 1 that with the exception of physical activity very little changed in the principals' perception of NCD-related health issues amongst school staff or learners. Table 1 also reports on the perceptions of principals from both arms of the study regarding school and environmental factors that could influence health promotion program implementation. It is clear that principals in the two groups reported very similar conditions and expected barriers. Lack of financial resources for example was selected as the most important barrier by principals from both groups.

**Table 1:** Characteristics of the schools, pre and post intervention

		Intervention Schools (*n* = 8)		Control Schools (*n* = 8)	
		2008	2011	2008	2011
A	School health environment				
1	School food and nutrition environment				
	Schools with shops	6	7	5	6
	Schools with shops selling fruit/salads	1	3	1	1
	Vegetable gardens at schools	1	4	2	3
	Schools having nutrition-related policies	5	7	7	7
	Vendors selling near/at schools	2	2	3	3
2	School physical activity and sport environment				
	Principals’ considering facilities adequate	1	-	0	-
	School grounds considered clean and safe by research team	4	4	3	3
3	Staff Health				
	Principals’ perception of staff health (as one of the top three health problems for staff)				
	Lack of physical activity by staff	3	8	8	8
	Tobacco use by staff	2	2	0	1
	Unhealthy diet among staff	4	4	2	2
	Overweight among staff	5	6	5	6
4	Chronic diseases and diabetes awareness				
	Principals’ perception of NCDs as one of the top three problems for				
	Staff	5	4	6	7
	Parents	2	4	3	3
	Learners (overweight/obesity)	0	0	0	1
B	Factors to consider in programme development/implementation				
1.	Community and environmental factors				
	Parents’ substance abuse reported as a problem	7	-	7	-
	Poverty and unemployment in the community (to a great extent a problem)	8	-	7	-
	Crime and violence in the community in general (to a great extent a problem)	5	-	6	-
2.	Barriers to adopting health programmes (principals’ perceptions)				
	Too little time within the timetable	2	-	3	-
	Too many competing priorities	2	-	3	-
	Lack of capacity/training and availability of human resources	4	-	4	-
	Lack of financial resources	6	-	5	-
	Inadequate facilities	4	-	3	-
	Lack of interest/willingness from outside organizations	1	-	1	-
	Lack of interest from learners	0	-	1	-
	Lack of interest/support from educators	1	-	0	-
	Lack of interest/support from parents	2	-	3	-
	Unsafe for learners to stay after school to participate	1	-	0	-

**Table 2:** Implementation support offered to and taken up by the eight HK intervention schools (2009 – 2011)

Implementation support offered by the research team	Uptake by schools		
	2009 (*n* = 8)	2010 (*n* = 8)	2011 (*n* = 8)
Action Planning Process (APP):			
*APP and implementation training workshops*	6		
*APP champions’ workshop*	8		
*Principals’ events*		5	
Physical activity:			
*Rope skipping*	3		
*Demonstration of PA lessons*	4		
*Physical activity training workshop*			1
*Physical activity road show*			3
Nutrition activities:			
*Food Based Dietary Guidelines workshop*			8
Healthy lifestyle curriculum:			
*Workshop*		5	
Staff Health:			
*Health monitoring and feedback/counselling*		8	8
Total uptake	21(32)	18(24)	20 (32)
Percentage uptake	65.6 %	75 %	62.5 %

The uptake by schools of intervention support in the form of structured activities is presented in Table 2. While all school champions attended the off-site champion workshop, only six of the schools elected to send staff to the APP and implementation workshop. The uptake of physical activity-related support was poor, with staff from only one school attending the off-site physical activity training workshop offered by experts in the field. A curriculum workshop, offered during a school holiday, with a monetary incentive offered to maximize attendance, was attended by staff from only five schools. However, a nutrition education workshop offered to schools in 2011, either off-site or at the school, was attended by all schools. Staff at all schools attended health promotion sessions at the schools in 2010 and 2011. Overall uptake of supporting events offered by the research team was 65.6 % in 2009, 75 % in 2010 and 62.5 % in 2011.

Table 3 presents a summary of the number of actions planned by the eight schools and those actually undertaken over the three-year period. School food and nutrition scored the highest, with 55.5 % of actions that were planned actually being undertaken. This was followed by chronic diseases and diabetes awareness with a 54.2 % uptake, school physical activity and sport environment with 25.9 %, and the staff health action area with only 20 % of actions being undertaken over the three years. While the total number of actions planned in the last year of the intervention did not increase from the previous year, the percentage of actions that were actually implemented increased substantially from 24 to 54 %. Only four of the actions reported in Table 3, of which only three were implemented, involved parents.

**Table 3:** Planned and actual actions by the eight HK intervention schools (2009 – 2011)

	School food and nutrition environment		School physical activity and sport environment		Staff health		Chronic diseases and diabetes awareness				
	PA^a^	AA^b^	PA	AA	PA	AA	PA	AA	Total PA	Total AA	% AA
2009	2	2	1	0	1	0	1	1	5	3	60
2010	21	7	20	3	0	0	13	3	54	13	24
2011	13	11	6	4	4	1	10	9	46	25	54
Total (ave)^c^	36	20 (2.5)	27	7 (0.9)	5	1 (0.1)	24	13 (1.6)			
% Actions Undertaken		55.5		25.9		20		54.2			

^a^PA - Planned actions

^b^AA - Actions actually undertaken

^c^Average per school over three years

More specific information is provided regarding the actions relating to the school food and nutrition environment in Table 4. Very few actions were planned in 2009 – only two for all eight schools. While this increased to 21 actions in 2010, only seven were actually carried out. In 2011, 13 actions were planned and 11 implemented, representing 84.6 % uptake. Most actions were around school policy, tuck shops, vendors, nutrition education and vegetable gardens. No activities were held regarding food at special events or food provided as a reward.

**Table 4:** School food and nutrition environment: actions at the eight HK intervention schools (2009 – 2011)

	2009		2010		2011		
	Planned actions	Actual actions	Planned actions	Actual actions	Planned actions	Actual actions	Total actual actions
Nutrition policy	1	1	8	1	2	1	3
School shops improved	1	1	6	2	3	3	6
Vendors dealt with	0	0	2	2	1	1	3
Food at special events	0	0	0	0	0	0	0
Lunch boxes	0	0	0	0	1	1	1
Food as reward	0	0	0	0	0	0	0
Nutrition education	0	0	2	0	2	2	2
School feeding	0	0	0	0	1	1	1
Vegetable gardens	0	0	3	2	3	2	4
Total %	2	2 (100 %)	21	7 (25.9 %)	13	11 (84.6 %)	20/36 55.5 %

The physical activity action area was very poorly represented with regards to planned and undertaken activities, as indicated in Table 5. No activities were undertaken in 2009; only three were undertaken in 2010 and four in 2011. This meant that some schools did not plan any actions. Overall, only seven physical activity actions took place at the eight schools over a three-year period. These were mainly related to lessons and improving sports. Only one staff health event took place during the intervention period and this related to physical activity behaviour (Table 6). Numerous activities took place in the NCDs awareness action area (Table 6) and all schools held a diabetes awareness day in 2011.

**Table 5:** School physical activity and sport environment: actions at the eight HK intervention schools (2009 – 2011)

	2009		2010		2011		
	Planned actions	Actual actions	Planned actions	Actual actions	Planned actions	Actual actions	Total actual actions
Policy introduced	0	0	1	0	0	0	0
Activity at break times	0	0	5	1	1	0	1
PA lessons	0	0	6	1	1	3	4
Improved sport	0	0	4	1	2	1	2
Family events	1	0	4	0	2	0	0
Total	1	0 (0 %)	20	3 (15 %)	6	4 (66.6 %)	7/27 26 %

**Table 6:** Staff health and NCD awareness: actions at the eight HK intervention schools (2009 – 2011)

	2009		2010		2011		
	Planned actions	Actual actions	Planned actions	Actual actions	Planned actions	Actual actions	Total actual actions
Staff health							
Staff health awareness	1	0	0	0	0	0	0
Food behaviour	0	0	0	0	2	0	0
PA behaviour	0	0	0	0	2	1	1
Role modelling	0	0	0	0	0	0	0
Total	1	0 (0 %)	0	0 (0 %)	4	1 (25 %)	1/5 20 %
NCD awareness							
Lesson plans	0	0	0	0	0	0	0
Posters	0	0	5	0	1	0	0
Learner home activities	0	0	1	3	0	0	3
Awareness days	1	1	7	0	8	8	9
Health checks	0	0	0	0	0	1	1
Parent talks	0	0	0	0	1	0	0
Total	1	1 (100 %)	13	3 (23 %)	10	9 (90 %)	13/24 54.2 %

Table 7 presents the qualitative findings of the post-intervention interviews with school staff at various levels. Far more benefits of the HK programme than barriers were identified during these interviews. The three most important themes that emerged from the data regarding perceived benefits were curriculum-related benefits, improvements in the school environment (mostly improvements to the schools’ tuck shops) and perceived beneficial effects on staff health. Direct benefit of the programme to children (mostly unspecified) and opportunities for parent engagement was raised to a lesser degree.

**Table 7:** Implementation barriers and benefits and reflections on the intervention as reported by school staff

Themes identified	Supporting quotations
**Perceived benefits**	
Curriculum	“The way it is set out man, it is simplified and it is not like an encyclopaedia where you have to search and do research, it is all there, like if you need information you don’t need any other resources. You can just use that and it will give you ample information.” *(Educator LO head)*
School environment improved	
Health of school staff	Yes, especially for me personally it made a big difference. Because every time I come here to weigh myself and I find out I am risk it worries me and I think I must reduce my weight and I must look at what I eat. Also my heart, my high blood pressure it went down I think because of my diet especially because I do not eat red meat anymore now I cut red meat it helped for me. *(Female educator, urban school)*
**Implementation Barriers**	
School environment and resources	“The biggest problem is that we have very little space. Not only our premises, the whole area [or] space is a problem here in…” *(Principal, rural school)*
Parents and socio-economic environment	“And then the environment in which we live plays a large role in what they [children] eat. They have to eat what they get… because there is little money for them to make changes to their diet. If it’s bread and coffee there is not much you can do about it.” *(Educator, urban school)*
Time pressures and human resources	“I think because sometimes we are too busy, like I think last year there were things happening, like, teachers that are going to have training sessions.” *(Educator, urban school)*
**Reflections on the intervention**	
More structured/focused/higher intensity approach needed	if we had somebody who can come over and show us how to do it, because we needed gardening and we need the soil and that will also help us be responsible maybe, because now we should have now you know, say we have this but we couldn’t take care of it I think it’s things like that, I think one project, if we have just one project, because that would have been a project that would stay here at school understand, it’s things like that, it’s things we can do and see them next time I think that would be very wise. *(Educator, urban school)*
Importance of champion/principal/ management	Moderator: “You said it is a (school name) project, yes? How would one get buy-in from the staff? How do you get the staff to…?”
	“Through the principal, the principal is the one where the buck stops. You should shake him up, you just contact him, [and] he shakes us up. (Laughing) (*Group of educators, urban school*)
More parent involvement/education	“The planning, yes, from our side the planning was very poor, definitely; and I think it can work the way you put it. If the parents and the School Governing Body are involved as well as most of the school teachers, because … and I cannot do everything, it’s impossible.” *(Educator and tuck shop coordinator, rural school)*

The themes relating to barriers that school staff experienced in implementing the HK programme (action planning and actions) at their schools are also shown in Table 7. Aspects related to the physical environment of the school and resources available in the school environment were most often mentioned as barriers. These were closely followed by parent and socio-economic-related issues. The participants, although less frequent, also experienced challenges regarding time, human resources and a lack of buy-in from school staff.

Participants were encouraged to share their feelings about the HK programme during the post-intervention interviews, and in Table 7, some of the most important themes emerging from these reflections are presented. Participants felt that a more structured and higher intensity approach would have been better. They also reflected on the important role of the champion and the management structure of the school for a programme such as HK to be successful. The need for greater involvement and education of parents was also an aspect that was raised.

## Discussion

The HK programme aimed to guide schools to plan and execute actions relevant to their school that would improve the schools’ physical activity and nutrition environments as well as the health of learners, school staff and parents. The results of this process, while varying over schools, were in general disappointing. Although a number of actions were decided upon every year, few were actually undertaken. Schools managed to implement 2.5 activities in the food and nutrition action area over the intervention period. Notwithstanding the greater awareness by principals of a lack of physical activity as a health priority for staff, less than one activity was implemented per school over the three-year period in the physical activity and sport environment and staff health action areas. Although these numbers only reflect the direct actions resulting from the intervention, and while there could have been other effects such as a greater emphasis on nutrition and physical activity teaching in the classroom, none of the schools had action plans that contained all components, including parental and community involvement, that have been identified as necessary for effective school-based programmes [22].

Various factors within the school environment and from the broader community environment could have played a role in the poor execution of the action planning and the actions selected. School staff pointed to the inadequate physical school environment, lack of resources, parent and socio-economic issues as the main barriers encountered in the process. However, the action planning guide allowed for improvement to the school physical environment and provided guidance for actions within the limited resources available. Barriers mentioned could possibly have contributed to school staff perceiving problems as insurmountable. A lack of time, finances and resources have also been described as problematic to various degrees in other school interventions in South Africa and elsewhere [16, 17]. The lack of planning in the physical activity action area could be related to the inability to address the school physical environment and perceived lack of resources in the particular sample as it has been shown that it is feasible to successfully implement a physical activity intervention in a low-income school setting in South Africa [23].

Concerns about socio-economic conditions as a barrier to implementing healthy eating guidelines have been raised in another study in the Western Cape of South Africa [24, 25]. Similar perceptions expressed in this study could have influenced the willingness of school staff to plan nutrition-related actions involving the school tuck shop and fundraising activities, as these could have been considered unaffordable for learners and a threat to a small additional income for the school and the livelihood of vendors from the surrounding community. This notion was dispelled in one of the schools that successfully intervened in their school tuck shop, where children chose to spend some of their money on fruit without any loss of income to the school. Although in a Northern Hemisphere context, Wharton *et al.* [26] also suggested that it is possible for schools to sell healthier options without losing revenue. The findings suggest that more intensified efforts to assist and train school staff in executing actions to improve the school food environment in low-income and resource-poor schools are necessary. This is supported by the recommendation from participants that a more structured and focused approach would have yielded better results.

Despite guidelines for the involvement of parents in all actions areas, very few action plans included parents. A lack of parent involvement was, however, one of the main barriers identified by school staff at the end of the intervention. Poor parental involvement in schools, especially in low-income areas have previously been identified in South Africa [27, 28] and experiences of school staff with this phenomenon could have made them reluctant to attempt to engage parents. Authors in the education sphere [28, 29] have argued that school staff should receive training in engaging parents and parents should receive training about their supporting and developmental roles in schools. Our findings suggest that this is also relevant for health related activities at schools and that such training components should be considered for future school-based interventions. The ISHP [10] allow for community-based and school-based health teams to work together and engaging parents through these teams could be investigated in future work.

Factors outside the school environment and not mentioned by school staff in the post-implementation interviews could have influenced the propensity of school staff to effectively implement the APP. For example, the HK intervention was planned and implemented at a time when many changes were taking place in the school system. In 2011, the Department of Basic Education [DBE) introduced a new curriculum to replace the outcomes-based education curriculum for which the curriculum component of the HK programme was developed. Annual National Assessments were instituted in Grade 4, and these assessments found overall very poor levels of literacy and numeracy skills. These findings prompted the DBE to direct school staff to focus all efforts on improving these skills. Educators were directed to not engage with outside organisations in ‘learner contact time’. In practice this meant that training and all other intervention activities, such as the APP depended on the good will of the principal, since departmental policy only allowed interventions to take place after hours, on Saturdays or during school holidays.

Early in the intervention in 2009, educators went on a prolonged strike for better wages and hence many planned activities were cancelled. In 2010, the World Soccer Cup in Cape Town caused many disruptions in the City of Cape Town and also disrupted several regular programmes, including those at schools.

Many of these challenges may not be unique to the South African setting. Schools have been described as highly complex systems, which makes the successful delivery of complex, multicomponent health interventions a daunting task [30]. In an effort to create a sustainable programme that would not be seen as an additional burden, the HK research team, to a certain degree, expected school staff to serve as multicomponent programme planners and implementers. Although only schools where principals indicated that they would like to implement health promotion programmes and had some facilities to support the implementation of such a programme were included in the study, the perception of district level managers and the school principals that the schools were ready to implement the programme could have been overly optimistic. In a retrospective evaluation of factors that influenced the implementation of the CATCH (Coordinated Approach to Child Health) programme [31] it was found that the construct “readiness to change” was the best predictor for implementation of the programme by classroom teachers. The authors speculate that school administrators play a limited role in directly implementing health programmes and may therefore overestimate the readiness of school staff to implement such programmes. Assessing readiness to change in those staff members from whom most will be expected during the implementation of health-related programmes could possibly play an important role in ensuring implementation success.

In our study participants did show some indication that they were at least considering the need for action. Evaluation of the initial phases of the APP showed that educators’ perceptions about the programme were overwhelmingly positive and they felt that the programme goals were clear [14]. Evaluation findings however suggested that role players at the schools might not have fully grasped what was expected from them at the outset of the APP. This, together with the immediate demands made by the multiple challenges reported in school environments in South Africa, such as frequent curriculum changes [32], administrative burdens and limited resources [33] as well as disengaged teacher behaviour [34]; might have prevented participants from moving to the action phase of the stages of change [31] as is clear in the small number of planned actions actually executed. Prevailing social norms regarding risk factors of NCDS such as poor dietary behaviour and a lack of physical activity [35, 36], may also have contributed to the lack of urgency of school staff to identify, plan and undertake relevant actions.

There is evidence from different settings that training teachers to teach and promote nutrition and physical activity in the classroom could lead to positive change in behaviour of children [36]. Results of interventions in schools in low-income settings, with similar challenges as the HK schools, furthermore show that strong outside facilitation could lead to good planning and execution of actions [37]. Recommendations from participants for more involvement from the research team seem to suggest that one of the barriers to the effective implementation of the APP could have been insufficient facilitation and it is therefore possible that more intensive training of educators as facilitators and promoters of healthy lifestyle behaviours could have resulted in better implementation outcomes. While the low level of facilitation in HK was specifically chosen to allow investigation of whether a sustainable programme could be implemented in schools without additional human resources, our findings seem to suggest that schools do need additional support and facilitation for effective implementation of such programmes. Although the ISHP provide the ideal policy environment for providing such support, management and leadership infrastructure as well as resources (human and otherwise), have been identified as barriers to the effective implementation of the ISHP [38]. Shung-King [39] points to one of the important factors to address these barriers as “prioritising the interventions that can make the greatest difference to children’s health and well-being, within the current resource constraints, with due consideration of the role and responsibilities of each sector so as to foster the best implementation context”.

If achieving healthy school environments are considered as essential for the present and future health of South African children it is necessary that a programme such as the HealthKick programme becomes one of these priority interventions and that the necessary training and support are made available to schools by the different government sectors involved in the ISHP. Creating awareness and urgency around health promoting aspects of schools could furthermore be enhanced if the nutrition and physical activity related aspects of school environments investigated in the HealthKick programme could be included into the DBE’s Whole School Evaluation Programme [40].

### Limitations

The study suffered from several limitations. The study only included two of the seven educational districts in the Western Cape Province and the findings can therefore not be generalised to the whole province or to South Africa. Another limitation included the consultation process with stakeholders before the implementation started. Although care was taken by the research team to familiarise themselves with the school environment with a situational analysis of a representative number of schools and consultation with district level staff, consultation with school staff other than principals and provincial and national level stakeholders could have been improved. This would be an important aspect to strengthen future school-based research. Another limitation mentioned earlier was that the process evaluation was conducted by the implementation team and not by an external evaluation team.

## Conclusions

An evaluation of the HK implementation process showed variable uptake of intervention support actions offered to the schools by the research team and poor implementation of the APP, particularly in the areas of physical activity and staff health. The process was intended to be carried out in such a way that school staff were empowered to make decisions that were most relevant to their school and community, rather than have something imposed on them by the research team (*e.g.* a particular evidence-based strategy). The poor implementation of the intervention could possibly be the result of numerous factors including lack of parenteral involvement, lack of time, resources and finances on the part of the school, poor socio-economic conditions experienced by the learners and their families as well as policy changes in the broader school environment. Implementation of the APP actions however did improve over time and some improvement in especially the school food and nutrition environment especially regarding school tuck shops and vegetable gardens have been observed. These findings together with the reasonably good uptake of intervention actions offered by the research team suggest that training and assistance with implementation infrastructure by the DoE could help schools in resource poor environments to implement programs aimed at creating school environments that are conducive to healthy lifestyle behaviours. As the policy environment for the provision of these resources already exist in the ISHP, it is clear that not only do the barriers to the effective implementation of the ISHP need to be addressed, but advocacy is also necessary for the inclusion of programmes such as HealthKick as a priority area in the ISHP. Our study’s findings contribute some evidence to what would be required to achieve healthy school environments within the existing education structures.
